# Bayesian Genomic-Enabled Prediction as an Inverse Problem

**DOI:** 10.1534/g3.114.013094

**Published:** 2014-08-25

**Authors:** Jaime Cuevas, Sergio Pérez-Elizalde, Victor Soberanis, Paulino Pérez-Rodríguez, Daniel Gianola, José Crossa

**Affiliations:** * Colegio de Posgraduados, 56230, Montecillo, Texcoco, Edo. de México; † Universidad de Quintana Roo, Chetumal, Quintana Roo, México; ‡ Department of Animal Sciences, University of Wisconsin, Madison, Wisconsin 53706; § Biometrics and Statistics Unit, International Maize and Wheat Improvement Center (CIMMYT), 06600, México, D.F., México

**Keywords:** Bayesian regression, shrinkage, prior distribution, inverse regression, GenPred, shared data resources, genomic selection

## Abstract

Genomic-enabled prediction in plant and animal breeding has become an active area of research. Many prediction models address the collinearity that arises when the number (*p*) of molecular markers (e.g. single-nucleotide polymorphisms) is larger than the sample size (*n*). Here we propose four Bayesian approaches to the problem based on commonly used data reduction methods. Specifically, we use a Gaussian linear model for an orthogonal transformation of both the observed data and the matrix of molecular markers. Because shrinkage of estimates is affected by the prior variance of transformed effects, we propose four structures of the prior variance as a way of potentially increasing the prediction accuracy of the models fitted. To evaluate our methods, maize and wheat data previously used with standard Bayesian regression models were employed for measuring prediction accuracy using the proposed models. Results indicate that, for the maize and wheat data sets, our Bayesian models yielded, on average, a prediction accuracy that is 3% greater than that of standard Bayesian regression models, with less computational effort.

Predicting complex traits possibly affected by large numbers of genes (each one having a small effect) that are greatly affected by the environment is a difficult task. Information from dense molecular markers attempts to exploit linkage disequilibrium between at least one marker and at least one putatively causal locus, so as to predict the genetic values of individuals based on their phenotypic data.

There is a vast literature describing methods that use different functions of markers for predicting genetic values (*e.g.*, de los Campos *et al.* 2012; Gianola 2013), starting with the seminal work of Meuwissen *et al.* (2001), in which they propose genome-based prediction functions that regress phenotypes on all available markers by using a Gaussian linear model with three different prior distributions on marker effects. Several regularized regression models, such as ridge regression (Hoerl and Kennard 1970), the Least Absolute Shrinkage and Selection Operator (*i.e.*, LASSO) (Tibshirani 1996), and its Bayesian counterpart (Park and Casella 2008; de los Campos *e al*. 2010), have been described and used for genomic-based prediction in animals and plants (de los Campos *et al.* 2009, 2010; Crossa *et al.* 2010, 2011; Gónzalez-Camacho *et al.* 2012; Heslot *et al.* 2012; Pérez-Rodríguez *et al.* 2010, 2012).

The basic quantitative genetic model describes the *i*^th^ response or phenotype ($y_{i}$) as the sum of an unknown genetic value ($g_{i}$) plus a residual $\varepsilon_{i}$ expressed as a deviation from some general mean (μ); thus, the basic model is $y_{i}=g_{i}+\varepsilon_{i}$ (i=1,...,n). The unknown genetic value can be represented as a function of genotypes with a large number of genes that may involve all gene × gene interactions, if there are any. Because the genes affecting a trait are unknown, this complex function must be approximated by, for example, a regression of phenotype on marker genotypes. Large numbers of bi-allellic markers on $\left\{x_{i1},\ldots ,x_{ip}\right\}$ ($x_{ij}$ is the number of copies of one of the two alleles observed in the *i^th^* individual at the *j^th^* marker) may be used in a regression function for predicting the genetic value of the *i*^th^ individual. The regression can be formulated as $u\left(x\right)=u(x_{i1},\ldots ,x_{ip})$ such that the basic genomic model becomes $y_{i}=u_{i}+\varepsilon_{i}$, where $\varepsilon_{i}$ is a model error that may include errors due to unspecified environmental effects, imperfect linkage disequilibrium between markers and the actual loci affecting the trait, and unaccounted for gene × gene and gene × environment interactions. In several applications, u(x) is a parametric linear regression of the form $u\left(x_{i1},\ldots ,x_{ip}\right)=\overset{p}{\underset{j=1}{\sum }}x_{ij}\beta_{j}$, where $\beta_{j}$ is the substitution effect of the allele coded as ‘one’ at the *j^th^* marker.

The linear regression function on markers becomes $y_{i}=\overset{p}{\underset{j=1}{\sum }}x_{ij}\beta_{j}+\varepsilon_{i}$, or, in matrix notation,

y=Xβ+ε (1)

where ***X*** is the *n*×*p* incidence matrix, ***β*** is the vector of unknown marker effects, and ***ε*** is an *n*×1 vector of random errors, typically assumed to follow the normal distribution $N\left(0,I_{n}\sigma_{\varepsilon }^{2}\right),$ where $\sigma_{\varepsilon }^{2}$ is the random error variance component. If the vector of marker effects is assumed random with normal distribution $N(0,I_{p}\sigma_{\beta }^{2})$, where $\sigma_{\beta }^{2}$ is the variance of marker effects, then the genetic value of the individuals is u=Xβ with a variance-covariance matrix proportional to the genomic relationship matrix $G=XX^{'}/\overset{p}{\underset{j=1}{\sum }}2q_{j}(1-q_{j})$ (where $q_{j}$ is the frequency of allele “1” often estimated from the data at hand); this random effect leads to the genomic best linear unbiased predictor (Vanraden, 2007, 2008), which is extensively used in genomic-assisted prediction. Note that the Bayesian ridge regression (BRR) is equivalent to the genomic best linear unbiased predictor when the distribution of single-nucleotide polymorphism (SNP) effects is regarded as Gaussian with mean **0** and variance $I_{p}\sigma_{\beta }^{2},\text{that}\;\text{is}$ $N(0,I_{p}\sigma_{\beta }^{2})$ (Pérez-Rodríguez *et al.* 2012).

The regression function $u\left(x_{i1},\ldots ,x_{ip}\right)$ also can be represented by semiparametric approaches, such as reproducing Kernel Hilbert space regressions or different types of neural networks (Gianola *et al.* 2006, 2011; Gianola and Van Kaam 2008; de los Campos *et al.* 2010; González-Camacho *et al.* 2012; Pérez-Rodríguez *et al.* 2012). de los Campos *et al.* (2012) reviewed penalized linear regression models and Bayesian shrinkage estimation methods.

The basic linear model (1) is generally overparameterized and therefore ill-conditioned because there are many more predictors (markers) than individuals with observations (*p* > >*n*), as well as strong collinearity among predictors due to linkage disequilibrium; hence ***X*** is not a full column rank. These challenges can be overcome using what is called the inverse problem approach (Aster *et al.* 2005; Tarantola 2005). The discrete inverse problem approach for matrices of incomplete rank or that are ill-conditioned due to possible collinearity is discussed at length by Aster *et al.* (2005), whereas Tarantola (2005) addresses the continuous version of the same problem. Cavalier (2008) summarized the inverse problem as a nonparametric regression, $d=T\theta +\tilde{\varepsilon }$, of a transformed data vector ***d*** on the linear operator $T_{n\times n}$ with $\theta \in \Theta_{n}\subseteq {\mathbb{R}}^{n}$ as a vector of regression parameters and $\tilde{\varepsilon }$ as a vector of Gaussian errors. Within a Bayesian framework, Knapick *et al.* (2012) proposed a solution by using the singular value decomposition of ***T*** such that the decay of singular values is mimicked in the prior distribution of *θ*. Thus, estimating the unknown transformed marker effects of *θ* from this perspective seems of interest but has not been attempted in genome-enabled prediction. The inferential solution to the inverse problem is based on the posterior distribution of *θ*, with the variability of $\tilde{\varepsilon }$ and prior beliefs about *θ* conveyed through probabilistic models.

In the prediction context, de los Campos *et al.* (2010) used the fact that the matrix ***G*** (or ***X***) may be eigen-decomposed as $G=UDU^{\prime }$, with ***D*** containing the eigenvalues of ***G***, and ***U*** being its corresponding eigenvectors. This transformation allows reducing the original highly dimensional data to fewer dimensions by extracting most of the signals in the “first” components of the decomposition and relegating the noise to the “last” components. As de los Campos *et al.* (2010) demonstrated, the regression of phenotypes on all markers is equivalent to the regression of phenotypes on a set of rotated covariates. Implementing regression methods with p>>n generally requires either shrinkage estimation or reducing the dimensionality of the data, which is the approach considered here. Recently, Gianola (2013) studied the influence of the prior on the posterior inference of marker effects in the overparameterized models used in genome-enabled prediction. He concluded that a main driving force in the various Bayesian models (Bayesian alphabet) is the prior and not the data; thus different priors will produce different results mainly because their shrinkage effect varies.

In this work, we propose using the parametric model (1) within the framework of inverse problems and a Bayesian approach to predict genetic values of individuals when *p* > >*n*. This proposal is similar to that introduced by de los Campos *et al.* (2010) for dimension reduction. However, ours has several different features, including an orthogonal transformation of the observed data (***y****)*, as well as differential shrinkage as a result of the prior variance assigned to the regression parameters.

## Materials and Methods

### Statistical methods

In model (1), when ***X*** is not of full rank, the least squares solution for the unknown ***β*** is neither unique nor stable. These ill-conditioned problems may be analyzed within the framework of an inverse problem theory by using the singular value decomposition of ***X*,** of order *n*×*p* (Aster *et al.* 2005), as $X=\mathrm{US}V^{\prime },$ where ***U*** and ***V*** are orthonormal matrices of orders *n*×*n* and *p*×*p*, respectively, and ***S*** is a rectangular *n*×*p* matrix containing *n* non-negative singular values ordered from largest to smallest, $s_{1}\geq s_{2}\geq \cdots \geq s_{n}$. One can write $S=[S_{1},S_{2}]$, where $S_{1}$ is the n×n matrix with singular values along its diagonal, and $S_{2}$ is a matrix of order *n*×(*p-n*) and all its entries are null. Consider the linear transformation on both sides of (1):

$$U^{\prime }y=U^{\prime }\mathrm{US}V^{\prime }\beta +U^{\prime }\varepsilon$$

and let $d=U^{\prime }y$, $b=V^{\prime }\beta$ and $\tilde{\varepsilon }=U^{\prime }\varepsilon$. Since $U\prime U=I_{n}$, the model (1) becomes:

$$d=\mathrm{Sb}+\tilde{\varepsilon }$$ (2)

In (2), ***d*** and $\tilde{\varepsilon }$ are vectors each of order *n*×1 and ***b*** is a vector of order *p*×1. The column vector ***b*** is partitioned as $b=\left[\begin{array}{c} b_{1}\\ b_{2} \end{array}\right],$ where $b_{1}$ is an *n*×1, and $b_{2}$ has order (*p-n*)×1. Therefore, equation (2) becomes:

$$d=S_{1}b_{1}+S_{2}b_{2}+\tilde{\varepsilon }=S_{1}b_{1}+\tilde{\varepsilon }$$

because $S_{2}b_{2}$ is zero for any value of $b_{2}$. Here only the first *n* entries of ***b*** can be inferred.

Since $\tilde{\varepsilon }=U^{\prime }\varepsilon$, it follows that $\tilde{\varepsilon }=U^{\prime }\varepsilon \sim N\left(0,U\prime U\sigma_{\varepsilon }^{2}\right)=N(0,I_{n}\sigma_{\varepsilon }^{2})$. The distribution of the transformed data ***d***, given ***b*** and $\sigma_{\varepsilon }^{2},$ is

$$f(d|b,\sigma_{\varepsilon }^{2})=\overset{n}{\underset{i=1}{\prod }}N(d_{i}|s_{i}b_{i},\sigma_{\varepsilon }^{2}\text{)}$$

where $d_{i}$ is the *i-th* element of **d** and has the form

$$d_{i}=s_{i}b_{i}+{\tilde{\varepsilon }}_{i},\;(i=1,\ldots ,n$$) (3)

since $S_{1}=\text{diag}\{s_{1},\ldots ,s_{n}\}$. To recover the original parameter ***β***, the p×p matrix **V** is partitioned into $V=[V_{1},V_{2}]$, where $V_{1}$ contains the first *n* columns of ***V***, and $V_{2}$ contains the remaining p−n, so that $\beta =V_{1}b_{1}+V_{2}b_{2}$. The *p* original parameters are recovered as $\beta =V_{1}b_{1},$ whereas $b_{2}$ does not contribute to fitting the data, that is:

$$\beta =\overset{n}{\underset{i=1}{\sum }}b_{i}V_{i,1}$$ (4)

where $V_{i,1}$ is the *i-th* column of $V_{1}$. Hence

$$cov\left(\beta |b_{2}=0\right)=V_{1}cov\left(b_{1}\right)V_{1}^{\prime }$$ (5)

and, for example, independent Gaussian priors can be assigned to each of the elements of $b_{1}$. Obviously, $b_{2}$ is an unknown quantity, so a prior must be assigned to this vector. However, as shown previously and in Gianola (2013), the likelihood function does not depend on $b_{2}$, so the data do not provide information about $b_{2}$. Here, the marginal posterior of $b_{1}$ is the likelihood times its prior, and the marginal posterior of $b_{2}$ is equal to its prior. Thus, if the Dirac delta function is assigned as a prior to each element of $b_{2}$, we get (4) as the quantity of interest and can safely leave $b_{2}$ out of the analysis. Thus $b_{1}$, $V_{1}$ and $S_{1}$ will herein after be denoted as ***b***, ***V*** and ***S***, respectively, without loss of generality.

The ordinary least squares estimator of ***β*** found using the generalized Moore-Penrose inverse method is

$$\beta^{*}=\overset{n}{\underset{i=1}{\sum }}\frac{d_{i}}{s_{i}}V_{i}$$ (6)

Thus, $\beta^{*}$ depends on the ratios $\frac{d_{i}}{s_{i}},i=1,\ldots ,n,$ between transformed data points and the decaying singular values, which could be interpreted as representing a decreasing signal-noise ratio sequence. So, as singular values decay, there is an increase in the noise component of the least squares estimate of component ***β***. This signal-noise ratio is inversely proportional to the singular values, as is clearly shown by the expression:

$$\frac{d_{i}}{s_{i}}=b_{i}+s_{i}^{-1}{\tilde{\varepsilon }}_{i}$$ (7)

The least squares estimator of $b_{i}$ can be computed from expression (7) as $b_{i}^{*}=\frac{d_{i}}{s_{i}}$ and serves as a basis for regularizing estimation through truncation or weighting.

### A Bayesian inverse regression model

Several methods have been proposed for mitigating ill-conditioned regression problems by shrinking the solution space via restricting the magnitudes of the estimates and their variance. One of the first proposals was the ridge regression estimator (Hoerl and Kennard 1970). Since model (3) has the form $d=\mathrm{Sb}+\tilde{\varepsilon }$, with $\tilde{\varepsilon }∼N(0,I_{n}\sigma_{\varepsilon }^{2})$, the ridge regression estimator is:

$$b_{\gamma }={\left(S^{\prime }S+\Gamma \right)}^{-1}S^{\prime }d={\left(S^{2}+\Gamma \right)}^{-1}S\text{d}$$ (8)

with ***Γ*** as a diagonal matrix of dimensions *n*×*n* with values on the diagonal. Here γ>0 is a vector of parameters that reduce ill-conditioning. If their magnitude increases excessively, this can lead to a poor fit of the model to the data. Therefore, the choice of vector ***γ*** is critical.

Hoerl and Kennard (1970) showed that there is a range of ***γ*** values where the mean squared error (MSE) of estimates is smaller than the corresponding MSE of the ordinary least squares solution. They minimized MSE by choosing

$$\gamma_{i}=\frac{\sigma_{\varepsilon }^{2}}{b_{i}^{2}},\;\left(i=1,\ldots ,n\right)$$ (9)

This requires knowing the true values of ***b***. However, (9) can be used to justify the choice

$$\gamma \approx \frac{n\sigma_{\varepsilon }^{2}}{{\hat{b}}^{\prime }\hat{b}}\approx \frac{{\hat{\sigma }}_{\varepsilon }^{2}}{{\hat{\sigma }}_{b}^{2}}$$ (10)

where $\hat{b}$, ${\hat{\sigma }}_{b}^{2}={\hat{b}}^{\prime }\hat{b}/n$, and ${\hat{\sigma }}_{\varepsilon }^{2}$ are estimates of ***b***, $\sigma_{b}^{2}$,$\;\text{and}\;\sigma_{\varepsilon }^{2}$, respectively, thus providing a single (global) shrinkage parameter.

In the Bayesian approach, the prior distribution reflects prior beliefs about $b_{i}$, and its variance affects the extent to which the least squares estimate moves toward the prior mean. To construct a model along the lines of de los Campos *et al.* (2012), we adopted as the conditional prior distribution of $b_{i}$

$$p\left(b_{i}|\lambda_{i}\right)=N\left(b_{i}|0,\lambda_{i}\right),\;i=1,\ldots ,n$$ (11)

where the $b_{i}$ coefficients (given $\lambda_{i}$) are conditionally independent. Therefore, the joint posterior distribution of ***b*** and $\sigma_{\varepsilon }^{2}$ in model (3) is given by:

$$p\left(b,\sigma_{\varepsilon }^{2}|d,S,\lambda \right)\propto \left\{\overset{n}{\underset{i=1}{\prod }}N\left(d_{i}|s_{i}b_{i},\sigma_{\varepsilon }^{2}\right)N\left(b_{i}|0,\lambda_{i}\right)\right\}p\left(\sigma_{\varepsilon }^{2}\right)$$ (12)

where $\lambda_{i}$ (*i = 1,…,n*) are variance parameters and $p\left(\sigma_{\varepsilon }^{2}\right)$ is the prior distribution of $\sigma_{\varepsilon }^{2}$. Usually the conjugate prior for $\sigma_{\varepsilon }^{2}$ is a scaled inverse χ^2^ distribution with $\upsilon_{e}$ degrees of freedom and scale parameter $Sc_{\varepsilon }$, that is,

$$p\left(\sigma_{\varepsilon }^{2}\right)=\chi^{-2}(\sigma_{\varepsilon }^{2}|\upsilon_{\varepsilon },Sc_{\varepsilon })$$ (13)

From (12), the conditional posterior distribution of ***b*** is:

$$p\left(b|d,S,\lambda ,\sigma_{\varepsilon }^{2}\right)=\overset{n}{\underset{i=1}{\prod }}N\left(b_{i}|\frac{\lambda_{i}s_{i}d_{i}}{\lambda_{i}s_{i}^{2}+\sigma_{\varepsilon }^{2}},\frac{\sigma_{\varepsilon }^{2}\lambda_{i}}{\sigma_{\varepsilon }^{2}+\lambda_{i}s_{i}^{2}}\right)$$ (14)

Expression (14) indicates that the conditional posterior mean depends on the data ($d_{i}$), whereas the variance is a fixed but unknown quantity.

The conditional expectation of ***b*** in (14) can also be expressed as

$$E\left(b_{i}|d_{i},s_{i},\lambda_{i},\sigma_{\varepsilon }^{2}\right)=\frac{\lambda_{i}s_{i}d_{i}}{\lambda_{i}s_{i}^{2}+\sigma_{\varepsilon }^{2}}=f_{i}b_{i}^{*}$$

where $f_{i}=\frac{\lambda_{i}s_{i}^{2}}{\lambda_{i}s_{i}^{2}+\sigma_{\varepsilon }^{2}}$ is known as the ‘weighting factor’ that weights the least squares estimate $b_{i}^{*}=\frac{d_{i}}{s_{i}}$.

Because the magnitude of variance of $b_{i}^{*}(i=1,\ldots ,n)$ grows with *i*, Casella (1985) suggested applying a lower weighting factor for estimates with larger variance. The weighting factor assigns weights close to one to the first elements of $b^{*}$, whereas the remaining elements receive lower weights, and can take on values close to zero, or zero itself.

### Bayesian inverse parametric regression models

Four versions of the Bayesian inverse regression models are described in this section. The first two, Bayesian inverse ridge regression (BIRR) and Bayes A inverse (BAI), are versions of the standard BRR and Bayes A (Meuwissen *et al.* 2001), respectively. In these models, the decay of the prior variances is not explicitly considered, whereas the other two proposed models do incorporate a prior decay of the variances.

#### Bayesian inverse ridge regression (BIRR):

The posterior expectation of $b_{i}$, given $d_{i},s_{i},\lambda_{i}$ and $\sigma_{\varepsilon }^{2}$ in (14), can also be written as:

$$E\left(b_{i}|d_{i},s_{i},\lambda_{i},\sigma_{\varepsilon }^{2}\right)={\left(s_{i}^{2}+\frac{\sigma_{\varepsilon }^{2}}{\lambda_{i}}\right)}^{-1}s_{i}d_{i}$$

which agrees with the ridge estimator in (8). A special case (BIRR) is when all prior distributions of $b_{i}$ are assigned a constant variance $\lambda_{i}=\sigma_{b}^{2}$. Then

$$p\left(b_{i}|\lambda_{i}\right)=N\left(b_{i}|0,\lambda_{i}=\sigma_{b}^{2}\right)\;i=1,\ldots ,n$$

If a scaled inverse χ^2^ distribution is assigned to $\sigma_{b}^{2}$, the joint posterior distribution of $\left(b,\sigma_{b}^{2},\sigma_{\varepsilon }^{2}\right)$ is given by:

$$p\left(b,\sigma_{b}^{2},\sigma_{\varepsilon }^{2}|d,S,\upsilon_{b},Sc_{b},\upsilon_{e},Sc_{e}\right)\propto \{\overset{n}{\underset{i=1}{\prod }}N\left(d_{i}|s_{i}b_{i},\sigma_{\varepsilon }^{2}\right)N\left(b_{i}|0,\sigma_{b}^{2}\right)\}\chi^{-2}\left(\sigma_{b}^{2}|\upsilon_{b},\upsilon_{b}Sc_{b}\right)p(Sc_{b})\chi^{-2}(\sigma_{\varepsilon }^{2}|\upsilon_{\varepsilon },Sc_{\varepsilon })$$ (15)

Note that model BIRR is analogous to the standard BRR in the sense that they both assign to the regression parameters a prior distribution with mean zero and constant variances.

#### Bayes A inverse (BAI):

Bayes A (Meuwissen *et al.* 2001) assigns the scaled *t* distribution as a prior to each marker effect, which can be represented as a normal-gamma mixture. We then have a hierarchical model with two levels: in the first one, a normal distribution with zero mean and variance $\lambda_{i}=\sigma_{b_{i}}^{2}$ is used as a conditional prior for ***b*** and, in the second level, the same scaled inverse χ^2^ prior distribution is used for all $\sigma_{b_{i}}^{2}$ (Yi and Xu 2008). Thus the joint posterior distribution of $(b,\sigma_{b_{i}}^{2},Sc_{b},\sigma_{\varepsilon }^{2}$) is

$$p\left(b,\sigma_{b_{1}}^{2}\ldots ,\sigma_{b_{n}}^{2},Sc_{b},\sigma_{\varepsilon }^{2}|d,S,\upsilon_{b},Sc_{b},\upsilon_{\varepsilon },Sc_{\varepsilon }\right)\propto \{\overset{n}{\underset{i=1}{\prod }}N\left(d_{i}|s_{i}b_{i},\sigma_{\varepsilon }^{2}\right)N\left(b_{i}|0,\sigma_{b_{i}}^{2}\right)\chi^{-2}\left(\sigma_{b_{i}}^{2}|\upsilon_{b},\upsilon_{b}Sc_{b}\right)\}p(Sc_{b})\chi^{-2}\text{(}\sigma_{\varepsilon }^{2}|\upsilon_{\varepsilon },Sc_{\varepsilon })$$ (16)

According to Yi and Xu (2008), when $\upsilon_{b}=0,\text{and}\;Sc_{b}=0$, this induces an improper distribution of ***b***. Therefore, these hyper-parameters should be greater than zero. We assigned $\upsilon_{b}=3$ and, for $Sc_{b}$, a uniform distribution with support on the interval (0,A), where A > 0. It should be noted that model BAI is not equivalent to the standard Bayes A because the prior variance of ***b*** under BAI is not the prior variance of the transformation $V^{\prime }\beta$ under Bayes A.

#### Bayesian inverse regression with decay model 1 (BIR1):

The mean of the posterior distribution of ***b*** can be seen as the product of a filter producing values that tend to shrink the least squares estimate toward 0 through the weighting factor $f_{i}$, which depends on singular values of ***X***. It is then reasonable to assume a prior that will shrink the values of $b^{*}$, in accordance with the singular values decay. Suppose we assign N(0,Ψ) as prior distribution to ***β*** with $\Psi =\mathrm{V\Lambda}V^{\prime }$ being the eigen-decomposition of ***Ψ***, where ***V*** is the right orthogonal p×p matrix of the singular value decomposition X=USV′. Then the prior distribution of ***b*** is N(0,Λ) because $b=V^{\prime }\beta$ and the vector $s^{2}$ of eigenvalues of $X^{\prime }X$ and the vector ***λ*** of eigenvalues of ***Ψ*** share the same ***V*** eigenvectors. Therefore, in this case it can be assumed that there is a relationship between $s_{i}$ and $\lambda_{i}$, i=1,…,n. Note that this relationship is consistent with what de los Campos *et al.* (2010) presented for semiparametric regression.

Knapick *et al.* (2012) proposed that the variances of the prior distribution be represented as $\lambda_{i}=\phi i^{-1-2\alpha }(i=1,\ldots ,n)$ in an attempt to mimic the decay of the singular values of ***X***, as suggested above. Thus, *ϕ* is a parameter that scales the rate $i^{-1-2\alpha }$ of decay of the prior variance with respect to *i*. Knapick *et al.* (2012) suggested fixing *α* and letting *ϕ* be the regularization parameter to be inferred, but without indicating how to do it. Since *ϕ* is an unknown scale parameter, it seems appropriate to assign an inverse scaled χ^2^ distribution to it with parameters $\upsilon_{\phi },Sc_{\phi }$. However, this may produce overshrinkage, which would be reflected in a poor fit and, thus, low prediction power. To address this problem, we redefined $\lambda_{i}$ as

$$\lambda_{i}=\phi \left(i^{-1-2\alpha }+h\right)$$ (17)

where *h* represents a smoothing parameter. The idea of adjusting the decay of a prior variance with a smoothing parameter was given by Maruyama and George (2011).

Thus the posterior density of $\left(b,\phi ,Sc_{\phi },\sigma_{\varepsilon }^{2}\right),$ given d,S, is:

$$p\left(b,\phi ,Sc_{\phi },\sigma_{\varepsilon }^{2}|d,S,h,\upsilon_{\phi },\alpha ,\upsilon_{\varepsilon },Sc_{\varepsilon }\right)\propto \{\overset{n}{\underset{i=1}{\prod }}N\left(d_{i}|s_{i}b_{i},\sigma_{\varepsilon }^{2}\right)N\left(b_{i}|0,\lambda_{i}\right)\}\chi^{-2}(\phi |\upsilon_{\phi },\upsilon_{\phi }Sc_{\phi })p(Sc_{\phi })\chi^{-2}(\sigma_{\varepsilon }^{2}|\upsilon_{\varepsilon },Sc_{\varepsilon })$$ (18)

where $p(Sc_{\phi })$ is a proper prior distribution for $Sc_{\phi }$.

#### Bayesian inverse regression with decay model 2 (BIR2):

A variant of BIR1 is obtained by defining $\lambda_{i}=\phi s_{i}^{\alpha }$. In this case, we may assign as prior for *ϕ* an inverse scaled χ^2^ distribution, with *α* kept fixed. Thus, the posterior is

$$p\left(b,\phi ,Sc_{\phi },\sigma_{\varepsilon }^{2}|d,S,\alpha \right)\propto \{\overset{n}{\underset{i=1}{\prod }}N\left(d_{i}|s_{i}b_{i},\sigma_{\varepsilon }^{2}\right)N\left(b_{i}|0,\phi s_{i}^{\alpha }\right)\}\chi^{-2}\left(\phi |\upsilon_{\phi },\upsilon_{\phi }Sc_{\phi }\right)p(Sc_{\phi })\chi^{-2}\text{(}\sigma_{\varepsilon }^{2}|\upsilon_{e},Sc_{e})$$ (19)

with $p(Sc_{\phi })$ being a proper prior distribution. When the value of *α* increases, shrinkage tends to increase, and when *α* decreases, shrinkage decreases and approximates BIRR when *α*=0. In the examples below, *α*=1 (the default value).

### Gibbs sampler

It is difficult to sample from the joint posterior distributions (15), (16), (18), and (19), because there are no known closed forms. However, it is possible to obtain the closed form for conditional distributions of the parameters (see the Appendix). This allows using Markov Chain Monte Carlo through the Gibbs Sampler (Gelfand and Smith 1990) algorithm, which samples sequentially from the full conditional distributions until it reaches a stationary process, converging with the joint posterior distribution.

We carried out convergence and diagnostic tests on different data sets. The Gelman-Rubin convergence tests for the four models were satisfactory, using lag-10 thinning results in low autocorrelations in each of the four models. The Raftery-Lewis test suggested a small burn-in and a number of iterations between 10,000 and 20,000 for the data set used below.

With the aim of decreasing the possible impact of Markov Chain Monte Carlo errors on prediction accuracy, we performed a total of 60,000 iterations with a burn-in of 10,000 and a thinning of 10 so that 5000 samples were used for inference.

## Experimental Data

### Maize data set

The maize data represented 21 trait-environment combinations for 300 tropical inbred lines genotyped with 55,000 SNPs each (González-Camacho *et al.* 2012). A first group of traits included female flowering or days to silking, male flowering (MFL) or days to anthesis, and the anthesis-silking interval. Each trait was evaluated under severe drought stress and in well-watered environments. This data set was also used by Crossa *et al.* (2010) for assessing prediction performance, but they used only 1148 SNPs.

In the second group of traits, grain yields (GYs) were obtained under severe drought stress and well-watered environments. Furthermore, GYs of the 300 maize lines also were measured in a large number of relatively high yielding environments and low yielding environments. In the third group of traits, the 300 maize lines also were evaluated in nine international environments for gray leaf spot (GLS), a disease caused by the fungus *Cercospora zeae-maydis*. Finally, in the fourth group, the same 300 lines were evaluated in another set of trials for northern corn leaf blight (NCLB), a disease caused by the fungus *Exserohilum turcicum*.

### Wheat data set

This data set included 306 elite wheat lines from CIMMYT’s Global Wheat Program that were also used by Pérez-Rodríguez *et al.* (2012). These lines were genotyped with 1717 diversity array technology markers generated by Triticarte Pty. Ltd. (Canberra, Australia; http://www.triticarte.com.au). Two traits were analyzed: days to heading, measured in 10 different environments, and GY, measured in seven different environments.

### Comparing models using cross-validation

Model predictions for each of the maize and wheat data sets were done in each of 50 random partitions, with 90% of the individuals in the training set and 10% of individuals in the testing set to be predicted. We used the same 50 random partitions as González-Camacho *et al.* (2012) and Pérez-Rodríguez *et al.* (2012). Pearson’s correlation between predicted and observed values and the predictive mean squared error (PMSE) were used as measures of predictive ability.

### Data and software

The 21 maize data sets and the 17 wheat data sets, as well as the R scripts developed to fit the predictive statistical models BIRR, BAI, BIR1, and BIR2 used in this study, are deposited at http://repository.cimmyt.org/xmlui/handle/10883/4036.

## Results

### Maize data sets

Table 1 shows mean correlations and mean PMSE from 50 random partitions for four models (BIRR, BAI, BIR1, and BIR2), along with the results of BRR, obtained using the BLR package in R (de los Campos and Pérez-Rodríguez 2010; Pérez-Rodríguez *et al.* 2010). The largest correlations and smallest PMSE for each trait-environment combination are in boldface. Models BIRR and BAI gave results similar to those of BRR. Models BIR1 and BIR2, which explicitly considered the decay of the singular values in the prior distribution, in general had greater average correlations and lower average PMSE, indicating better prediction ability than BRR, BIRR, and BAI.

**Table 1:** Maize data sets

Trait-Environment Combination^+^	Mean Correlation					Mean PMSE				
	BRR	BIRR	BAI	BIR1	BIR2	BRR	BIRR	BAI	BIR1	BIR2
FFL-WW	0.818	0.819	0.828	0.842	**0.847**	0.262	0.260	0.219	0.201	**0.196**
FFL-SS	0.754	0.755	0.759	0.762	**0.764**	0.342	0.325	0.325	0.324	**0.320**
MFL-WW	0.822	0.822	0.829	0.841	**0.850**	0.263	0.263	0.225	0.203	**0.198**
MFL-SS	0.776	0.777	0.784	0.782	**0.788**	0.318	0.318	0.299	0.298	**0.293**
ASI-WW	**0.582**	**0.582**	0.580	0.578	0.574	0.649	**0.648**	0.649	0.651	0.655
ASI-SS	0.613	0.614	**0.617**	0.614	0.611	0.653	0.652	**0.647**	0.649	0.650
Average	0.727	0.728	0.733	0.736	**0.739**					
% increase	−1.2%	−1.1%	−0.5%	0%	**0.4%**					
GY-SS	0.320	0.326	0.305	0.354	**0.360**	0.890	0.883	0.904	0.866	**0.862**
GY-WW	0.557	**0.558**	0.555	**0.558**	**0.558**	0.677	0.675	0.680	**0.674**	0.675
GY-HI	0.634	0.635	0.664	0.667	**0.674**	0.597	0.595	0.558	0.554	**0.546**
GY-LOW	0.410	0.412	0.419	**0.424**	**0.**423	0.863	0.845	0.847	**0.835**	0.837
Average	0.480	0.483	0.486	0.501	**0.503**					
% increase	−4.2%	−3.7%	−3.1%	0%	**0.4%**					
GLS 1	0.241	0.238	0.260	0.282	**0.287**	0.930	0.928	0.920	0.902	**0.900**
GLS 2	0.419	0.421	0.414	**0.427**	**0.426**	0.827	0.825	0.835	**0.819**	**0.819**
GLS 3	0.588	**0.589**	0.585	0.586	0.584	0.625	**0.624**	0.629	0.627	0.629
GLS 4	0.522	0.522	0.529	**0.533**	0.528	0.735	0.733	0.728	**0.721**	0.729
GLS 5	0.338	0.341	0.348	**0.360**	0.356	0.819	0.815	0.807	**0.801**	0.803
GLS 6	0.257	0.274	0.241	**0.278**	0.276	0.975	0.964	0.986	**0.959**	0.960
GLS 7	0.474	0.475	0.472	**0.480**	0.484	0.761	0.761	0.766	0.756	**0.750**
GLS 8	0.595	**0.596**	0.593	0.593	0.591	**0.618**	0.617	0.621	0.620	0.622
GLS 9	0.522	0.522	0.529	**0.533**	0.530	0.734	0.732	0.728	**0.723**	0.727
Average	0.442	0.442	0.441	**0.453**	0.445					
% increase	−2.9%	−2.4%	−2.6%	**0%**	−0.2%					
NCBL 1	0.649	0.649	**0.697**	0.693	**0.697**	0.592	0.592	**0.521**	0.527	0.523
NCBL 2	0.469	0.473	0.523	**0.526**	0.524	0.731	0.723	**0.673**	0.679	0.680
Average	0.559	0.561	0.61	0.610	**0.611**	0.656	0.658	0.646	**0.638**	0.639
% increase	−9.0%	−8.7%	0.2%	0%	**0.7%**					

Mean predicted correlation, average, % increase correlation with respect to model BIR1, and mean PMSE of five models, BRR, BIRR, BAI, BIR1, and BIR2, for 50 random partitions for each of 21 trait-environment combinations. The largest correlations and smallest PMSE for each trait-environment combination are in boldface. PMSE, predictive mean squared error; BRR, Bayesian ridge regression; BIRR, Bayesian inverse ridge regression; BAI, Bayes A inverse; BIR1, Bayesian inverse regression model 1; BIR2, Bayesian inverse regression model 2; FFL, female flowering; WW, well-watered environment; SS, severe drought stress; MFL, male flowering; ASI, MFL to FFL interval; GY, grain yield; HI, optimum environment; LOW, stress environment; GLS, *Cercospora zeae-maydis*; NCLB, *Exserohilum turcicum*.

Table 1 also shows average correlations and percentage differences from BIR1 for each group of trials: Flowering, GY, GLS, and NCLB. The approximate differences from BIR1 or BRR and BIRR were 4% for GY and 9% for NCLB, whereas the differences for GLS and Flowering were 2.6% and 1.2%, respectively. BIR1 and BIR2 had similar performance. BAI and BIR1 had similar results in the Flowering and NCLB groups, but BAI was slightly worse for GY and GLS. This finding indicates a 3% improvement in the overall average prediction ability of BIR1 and BIR2 over that of BRR, BIRR, and BAI. Model BIR1 had greater predictive correlations than BRR in 18 of the 21 trait-environment combinations, whereas BIR1 had smaller PMSE than BRR in 18 of the trait-environment combinations. Models BIR1 and BIR2 had the lowest PMSE in 16 of the 21 maize data sets. The PMSE of BIRR and BAI were similar.

Table 2 shows the number of times a model had a greater correlation than another model in 50 random partitions for each of the 21 trait-environment combinations. BIR1 was better than BIRR in 17 trait-environment combinations, *i.e.*, in 697 of 1050 comparisons. BIR1 was better than BAI in 16 trait-environment combinations, *i.e.*, in 669 partitions. Also, BIR2 was superior to BIRR and BAI in 640 and 610 partitions, respectively, of a total of 1050 random partitions.

**Table 2:** Maize data sets

Trait-Environment Combination	Number of Times One Model Had a Larger Correlation Than Another Model over 50 Random Partitions				
	BIR1 > BIRR	BIR1 > BAI	BIR2 > BIRR	BIR2 > BAI	BIR2 > BIR1
FFL-WW	38	32	40	35	28
FFL-SS	36	27	35	40	33
MFL-WW	41	36	41	38	33
MFL-SS	39	30	39	35	30
ASI-WW	24	23	19	16	16
ASI-SS	25	20	19	18	17
GY-SS	33	41	36	41	31
GY-WW	26	33	25	34	22
GY-HI	42	33	43	30	26
GY-LOW	32	29	31	27	25
GLS 1	35	33	37	33	29
GLS 2	31	34	30	33	19
GLS 3	21	28	18	26	20
GLS 4	32	29	28	23	17
GLS 5	33	32	31	29	21
GLS 6	26	32	26	32	21
GLS 7	34	37	31	34	31
GLS 8	18	24	19	23	24
GLS 9	30	29	29	24	24
NCBL 1	46	19	46	26	33
NCBL 2	46	15	44	21	23
Average	33	29	32	29	25

Number of times one model had a greater correlation than another model in 50 random partitions for each of the 21 trait-environment combinations. The models were BIRR, BAI, BIR1, and BIR2. BIR1, Bayesian Inverse regression model 1; BIRR, Bayesian inverse ridge regression; BAI, Bayes A inverse; BIR2, Bayesian inverse regression model 2; FFL, female flowering; WW, well-watered environment; SS, severe drought stress; MFL, male flowering; ASI, MFL to FFL interval; GY, grain yield; HI, optimum environment; LOW, stress environment; GLS, *Cercospora zeae-maydis*; NCLB, *Exserohilum turcicum*. Environment 1−9.

### Wheat data sets

Table 3 shows mean correlations of models BIRR, BAI, BIR1, and BIR2, along with results for models BL, BRR, Bayes A, and Bayes B, as reported by Pérez-Rodríguez *et al.* (2012) using the same 50 random partitions. The largest average correlation for each trait-environment combination is in boldface. The table also shows correlation standard deviations (in parentheses). In the case of trait days to heading, the average correlations for BIRR, BAI, BIR1, and BIR2 are very similar and better than those for BL, BRR, Bayes A, and Bayes B. Within the GY group, BIRR, BAI, and BIR1 have similar correlations; however, BIR2, BL, BRR, and Bayes B exhibit a smaller global average than BIRR, BAI, and BIR1. Table 4 shows the PMSE for both traits, and their values are in agreement with the results in Table 3.

**Table 3:** Wheat data sets

Trait	Environment	BL	BRR	Bayes A	Bayes B	BIRR	BAI	BIR1	BIR2
DTH	1	0.59 (0.11)	0.59 (0.11)	0.59 (0.11)	0.56 (0.11)	**0.60** (0.11)	0.60 (0.11)	0.60 (0.11)	0.59 (0.11
	2	0.58 (0.14)	0.57 (0.14)	0.61 (0.12)	0.57 (0.13)	0.62 (0.14)	0.61 (0.14)	0.62 (0.14)	**0.65** (0.12)
	3	0.60 (0.13)	0.60 (0.12)	0.62 (0.11)	0.60 (0.12)	0.62 (0.12)	0.62 (0.12)	0.62 (0.12)	**0.63** (0.12)
	4	0.02 (0.18)	**0.07** (0.17)	0.06 (0.17)	0.06 (0.17)	0.06 (0.18)	0.01 (0.18)	**0.06** (0.18)	0.04 (0.20)
	5	0.65 (0.09)	0.64 (0.10)	0.66 (0.09)	0.66 (0.09)	**0.65** (0.09)	**0.65** (0.09)	**0.65** (0.09)	**0.65** (0.09)
	8	0.36 (0.15)	0.37 (0.15)	0.36 (0.15)	0.35 (0.14)	0.38 (0.15)	**0.39** (0.15)	0.38 (0.15)	0.36 (0.15)
	9	0.59 (0.12)	0.59 (0.11)	0.53 (0.12)	0.52 (0.11)	**0.60** (0.11)	**0.60** (0.11)	0.60 (0.11)	0.57 (0.11)
	10	0.54 (0.14)	0.52 (0.14)	0.56 (0.13)	0.54 (0.14)	0.55 (0.13)	0.54 (0.14)	0.55 (0.13)	**0.56** (0.12)
	11	0.52 (0.15)	0.52 (0.16)	0.53 (0.13)	0.51 (0.13)	0.54 (0.15)	**0.54** (0.16)	0.54 (0.15)	0.52 (0.13)
	12	0.45 (0.19)	0.42 (0.18)	0.45 (0.18)	0.45 (0.18)	0.47 (0.18)	0.47 (0.18)	0.48 (0.18)	**0.49** (0.19)
	Average	0.49	0.49	0.50	0.48	0.51	0.50	**0.510**	**0.51**
	% increase	−4.0%	−4.3%	−2.6%	−5.8%	−0.1%	−1.1%	**0%**	**-0.7%**
GY	1	0.48 (0.13)	0.43 (0.14)	0.48 (0.13)	0.46 (0.13)	**0.48** (0.13)	**0.48** (0.14)	**0.48** (0.13)	0.47 (0.13)
	2	0.48 (0.14)	0.41 (0.17)	0.48 (0.14)	0.48 (0.14)	0.47 (0.15)	0.47 (0.15)	0.47 (0.15)	**0.48** (0.13)
	3	0.20 (0.21)	0.29 (0.22)	0.20 (0.22)	0.18 (0.22)	0.24 (0.22)	**0.25** (0.21)	0.24 (0.22)	0.21 (0.20)
	4	0.45 (0.15)	0.46 (0.13)	0.43 (0.15)	0.42 (0.15)	**0.46** (0.14)	**0.46** (0.14)	0.46 (0.14)	0.43 (0.14)
	5	0.59 (0.14)	0.56 (0.16)	0.75 (0.11)	0.74 (0.12)	0.58 (0.15)	0.58 (0.15)	0.59 (0.15)	0.58 (0.13)
	6	0.70 (0.10)	0.67 (0.11)	0.73 (0.08)	0.71 (0.08)	**0.71** (0.1)	**0.71** (0.10)	**0.71** (0.1)	**0.71** (0.1)
	7	0.46 (0.14)	0.50 (0.14)	0.42 (0.14)	0.40 (0.15)	**0.48** (0.14)	**0.48** (0.15)	0.48 (0.14)	0.42 (0.15)
	Average	0.48	0.474	**0.499**	0.484	0.489	0.49	0.489	0.473
	% increase	−1.7%	−2.9%	**2.1%**	−0.8%	0.2%	0.5%	0%	−3.5%

Mean predictive correlations (SD in parentheses) for GY and DTH by environment for eight models: BL, BRR, Bayes A, Bayes B (Pérez-Rodríguez *et al.* 2012), BIRR, BAI, and BIR1 and BIR2. Average correlation for each model and % increase correlation with respect to model BIR1 is shown. The largest values for each trait-environment combination are in boldface. DTH, days to heading; GY, grain yield; BL, Bayesian least absolute shrinkage and selection operator; BRR, RR-BLUP; BIRR, Bayesian inverse ridge regression; BAI, Bayes A inverse; BIR1, Bayesian inverse regression model 1; BIR2, Bayesian inverse regression model 2.

**Table 4:** Wheat data sets

Trait	Environment	BL	BRR	Bayes A	Bayes B	BIRR	BAI	BIR1	BIR2
DTH	1	13.02	13.18	12.72	13.23	12.81	12.85	12.8	**12.66**
	2	11.89	12.37	**10.65**	11.28	11.64	11.88	11.59	10.63
	3	8.18	8.44	**7.31**	7.59	8.09	8.04	8.08	7.44
	4	**21.59**	22.27	21.79	21.67	21.61	21.57	21.61	22.7
	5	8.86	9.23	8.48	8.37	8.78	8.81	8.78	8.68
	8	14.72	15.22	**14.54**	14.58	14.69	14.68	14.69	14.75
	9	21.38	21.44	23.71	23.93	21.36	21.40	**21.35**	22.22
	10	7.72	8.51	7.27	7.57	7.67	7.91	7.62	**7.27**
	11	6.83	7.12	**6.59**	6.74	6.78	6.74	6.77	6.77
	12	13.60	14.42	13.56	**13.46**	13.66	13.62	13.60	13.42
GY	1	0.07	0.09	0.07	0.07	**0.07**	**0.07**	0.07	0.07
	2	0.06	0.08	0.06	0.06	**0.06**	**0.06**	**0.06**	**0.06**
	3	0.06	0.07	0.06	0.06	**0.06**	**0.06**	**0.06**	**0.06**
	4	0.22	0.24	0.23	0.23	**0.22**	**0.22**	**0.22**	0.23
	5	0.39	0.44	**0.26**	0.27	0.40	0.40	0.40	0.39
	6	0.13	0.15	**0.12**	0.13	0.13	0.13	0.13	0.13
	7	**0.40**	0.41	0.43	0.44	**0.40**	**0.40**	**0.40**	0.43

Mean predicted mean squared error between observed and predicted values for GY and DTH of wheat lines in environments (1−11) for eight models: BL, BRR, Bayes A, Bayes B (Pérez-Rodríguez *et al.* 2012), BIRR, BAI, and BIR1 and BIR2. The smallest values for each trait-environment combination are in boldface. BL, Bayesian least absolute shrinkage and selection operator; BRR, RR-BLUP; BIRR, Bayesian inverse ridge regression; BAI, Bayes A inverse; BIR1, Bayesian inverse regression model 1; BIR2, Bayesian inverse regression model 2; DTH, days to heading; GY, grain yield.

## Discussion

The SVD transformation of the data generates a basic probability model such that the joint density of the data (given parameters) is the product of univariate independent random variables, $p(d|S,b,\sigma_{\varepsilon }^{2})=\overset{n}{\underset{i=1}{\prod }}N(d_{i}|s_{i}b_{i},\sigma_{\varepsilon }^{2}\text{)}$. Moreover, the new parameterization allows reducing the dimensionality of the vector of regression parameters from *p* to *n*. Because *p-n* parameters are not estimable, they do not contribute to data fitting and are considered to have a prior distribution with mean and variance equal to zero (a Dirac delta function). Because the proposed transformation yields *n* positive singular values, it reduces the number of parameters to *n*. This gives BIRR, BAI, BIR1, and BIR2 an extra computational advantage over BRR because the Gibbs sampler algorithm is faster due to the posterior distributions simulating a smaller number of parameters with univariate distributions. The proposed inverse Bayesian models implement the idea put forward by Casella (1985) that the increased instability of the OLS estimates $b_{i}^{*}$ can be ameliorated by decreasing the value of the weighting factor. Shrinkage is achieved using the weighting factor$\left(f_{i}\right)$, that is, $E\left(b_{i}|,d_{i},s_{i},\lambda_{i},\sigma_{\varepsilon }^{2}\right)=f_{i}b_{i}^{*}$, which weights the estimates $b_{i}^{*}$ such that the instability of $b_{i}^{*}$ is controlled with a smaller weighting factor. The weights are highly dependent on the singular values and on the variance of the $\lambda_{i}$ prior distribution.

In the following section, we describe the prediction performance of the four inverse Bayesian methods in terms of the magnitude and decay of the prior variance and the behavior of the OLS estimates $b_{i}^{*}$. Finally, we consider the pattern of noise amplification as a result of the decay of the singular values, which also depends on the data $d_{i}$.

### Shrinkage and prediction in the maize data set

**Figure 1:**
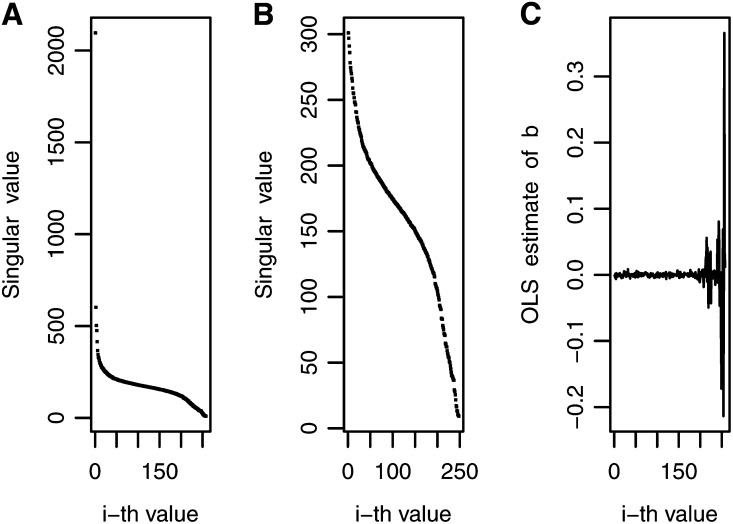
Decay of the singular values and noise pattern for maize trait-environment combination male flowering in well-watered environments: (A) decay of all singular values, (B) decay of all singular values except the first 10, and (C) noise in the ordinary least squares (OLS) estimates for all singular values.

For the 21 maize data sets, the ***X*** matrix had a rank equal to *n* with moderate decay in singular values, indicating moderate ill-conditioning. Figure 1, A−C shows the decay of singular values for data set MFL-WW. Figure 1A shows all singular values, and Figure 1B excludes the first 10 singular values and depicts the rapid decay in the first subsequent 45 singular values, a smaller rate of decay in singular values 45−200 and the rapid decay of singular values after the 200th singular value, reflecting the actual collinearity trend. Figure 1C shows that the OLS estimator of $b_{i}$ is very stable for the first 200 singular values and becomes very erratic at the end due to noise, coinciding with the rapid decrease in singular values after the first 200. In short, Figure 1, A−C show that more weight should be given to the first 200 least squares estimates ($b_{i}^{*})$ and less weight should be assigned to the last 50 $b_{i}^{*}$´s, which basically represent noise. The four inverse Bayesian methods had good predictive power, with differences being due to the variance assigned to the prior distribution of parameter $b_{i}$.

**Figure 2:**
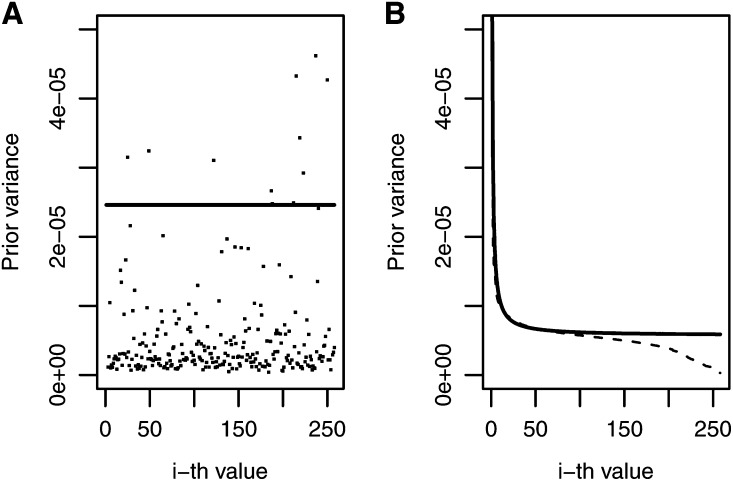
Maize trait-environment combination male flowering in well-watered environments: the prior variances for the *i*-th singular values for four inverse Bayesian regression models: (A) Bayesian inverse ridge regression (line) and Bayes A inverse (dots); (B) Bayesian inverse regression model 1 (solid line) and Bayesian inverse regression model 2 (dashed line).

Figure 2, A and B depicts the prior variance for models BIRR, BAI, BIR1, and BIR2 for trait-environment combination MFL-WW. In Figure 2A, the prior variance of $b_{i}$ for BIRR is represented by a solid line, whereas $\lambda_{i}$ used in BAI are scattered dots, each representing an individual. It is interesting to note that, for MFL-WW, most of the $\lambda_{i}$ values for BAI, BIR1, and BIR2 were smaller than those for BIRR, represented by a solid line. This indicates that BAI, BIR1, and BIR2 cause more shrinkage. Figure 2B depicts the decay of singular values for BIR2 (dashed line) and BIR1 (solid line), both mimicking the current decay of singular values shown in Figure 1, A and B. The decay of BIR1 reflects the polynomial function $i^{-1}$, but smoothed by the parameter *h*, as indicated in (18), with less shrinkage for the first singular values and increasing shrinkage toward the later singular values.

**Figure 3:**
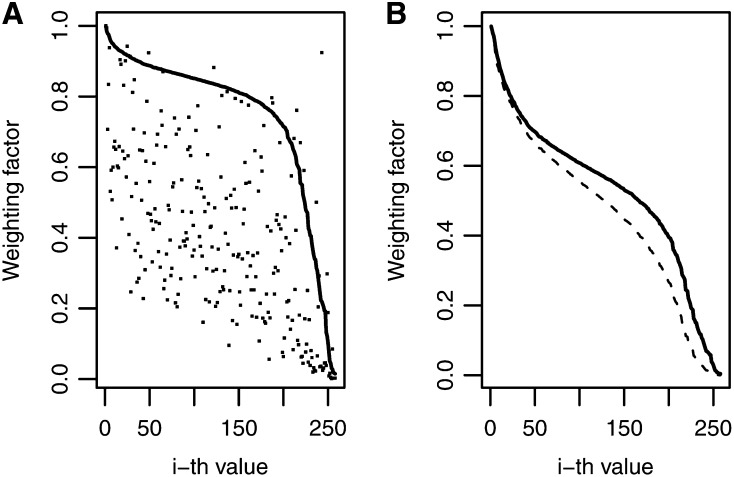
Maize trait-environment combination male flowering in well-watered environments: weighting factor values for four inverse Bayesian regression models: (A) Bayesian inverse ridge regression (solid line) and Bayes A inverse (dots); (B) Bayesian inverse regression model 1 (solid line) and Bayesian inverse regression model 2 (dashed line).

These prior variances are reflected in the weighting factor. For example, Figure 3A shows the weights for BAI and for BIRR and, in general, there is more shrinkage for BAI than for BIRR. Figure 3B shows that the weights for BIR1 are slightly larger than those for BIR2. When comparing Figures 2 and 3, it is clear that as shrinkage increases, the weights come closer to 0. The four inverse Bayesian methods assigned weights that parallel the decay of the singular values (Figure 1, A and B), but for trait MFL-WW, the methods that reduced the weighting factors the most were BIR2 and BIR1, which had larger mean predictive correlations (0.850 and 0.841, respectively) than BIRR, with a mean predictive correlation of 0.822 (Table 1). This may be a reflection of some overfit caused mainly by larger weights used in the last 50 estimators, where noise is concentrated (Figure 1C). Trends were different for other traits; for example, for trait-environment combination GLS-3, BIRR, BIR1, and BIR2 had mean predictive correlations of 0.589, 0.586, and 0.584, respectively.

**Figure 4:**
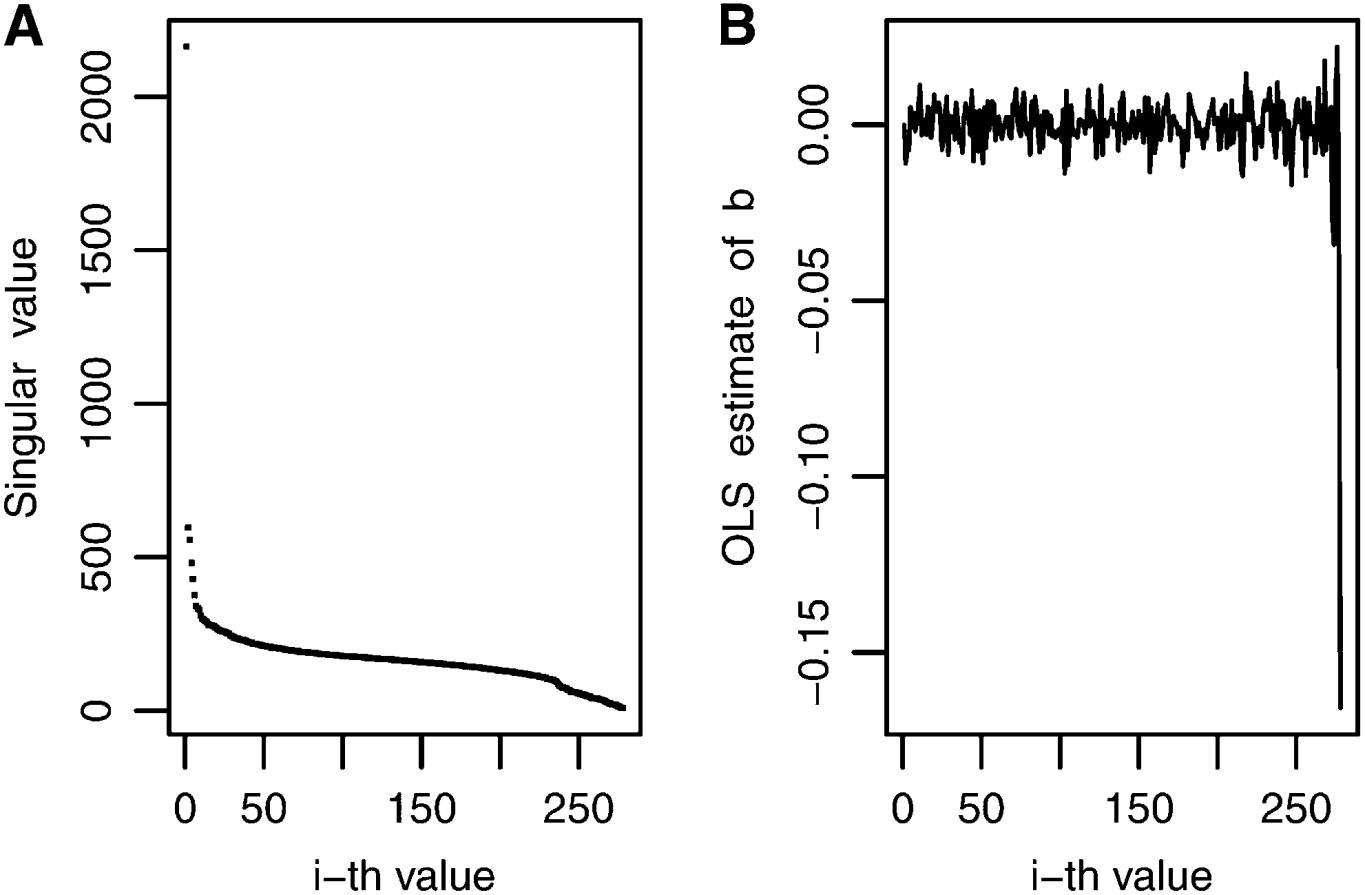
Decay of singular values and noise pattern for maize trait-environment combination gray leaf spot-3: (A) decay of all singular values; (B) noise in the ordinary least squares (OLS) estimates for all singular values.

Comparing Figure 1C (OLS estimates for MFL-WW) and Figure 4B (OLS estimates for GLS-3), instability of OLS estimates is observed for the last 50 OLS values in MFL-WW but this occurred in only a few of the last OLS estimates for GLS-3. This indicates that trait MFL-WW required more shrinkage than that needed for trait GLS-3. This may explain why, for MFL-WW, model BIR2, which showed more shrinkage than BIRR, had a better predictive correlation than BIRR. For the same reasons, model BIRR was a better predictor than BIR2 for trait GLS-3. BIR1 had a good predictive correlation for most traits because the decay of the variance was smoothed out by $h=\frac{1}{s_{r}}\sim 0.10$ (see Equation 17), which allowed “intermediate” shrinkage.

### Shrinkage and prediction in the wheat data set

**Figure 5:**
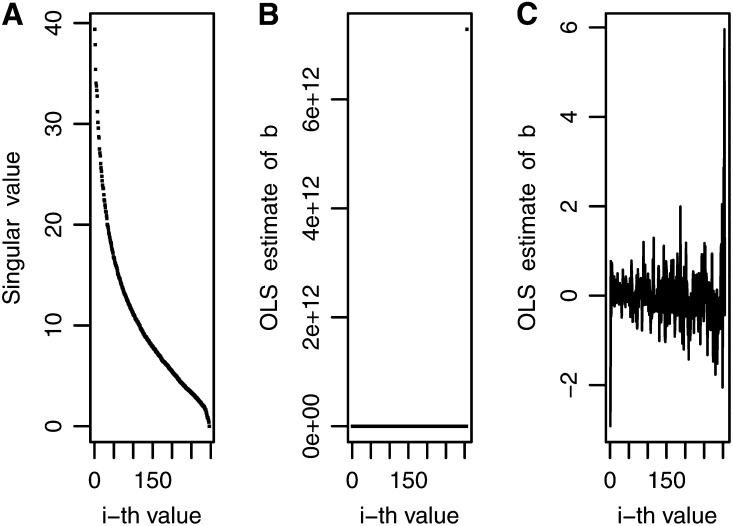
Decay of singular values and noise pattern for wheat trait-environment combination days to heading-1: (A) decay of all singular values except the first 10; (B) noise in the least squares estimates including when all singular values are included; (C) noise in the ordinary least squares (OLS) estimates for all singular values except the last one.

The decay pattern of singular values and the noise in the wheat data set were different from those found in the maize data set. The ***X*** matrix had a rank equal to *n*-1, indicating stronger collinearity and, thus, greater decay of singular values than that found in the maize data sets; in addition, data for various trait-environment combinations had outliers and were more complex. This is shown, for example, in Figure 5, A−C for trait-environment combination DHT-1. Figure 5A shows that the decay of singular values for this trait was more pronounced than that for MFL-WW in maize (Figure 1, A and B). This data set has more collinearity than the maize data, making it difficult to visualize and detect where random noise is manifested in the OLS estimates; this represents a moderate-to-high ill-conditioning situation. Figure 5B shows that one singular value close to zero (upper right-hand side of the figure) magnified the OLS estimate, but when that outlier was removed, the noise pattern was more clearly delineated (Figure 5C). Noise increased systematically toward the last singular values, where the signal was practically lost.

In cases with strong collinearity, Kyung *et al.* (2010) suggested ridge regression as an effective model. This may explain, in part, why BIRR, BAI, and BIR1 had similar performance, whereas BIR2, with more shrinkage than the other models, had a mean predictive correlation similar to those of other models but with differential performance for each trait-environment combination. The greater complexity of the ***X*** matrix, as well as of the phenotypes included in the wheat data, suggests that other prediction models, such as the semiparametric regression described by Pérez-Rodríguez *et al.* (2012), may be more suitable.

## CONCLUSIONS

Models were developed within the framework of inverse regression theory. Inverse solutions induce some parsimony while keeping conditional independence of the transformed phenotypes. Inverse solutions make it possible to visualize that noise is inversely proportional to singular values. The univariate structure allows graphical depiction of shrinkage behavior according to the prior variance and weighting factor. The models developed here seem to deal well with the ill-conditioning and random noise problems arising in genomic prediction, where *p* > >*n*.

The differences among models depend on several factors, such as the pattern and decay of singular values. It is expected that when the decay of the singular values is moderated, the prediction accuracy of the proposed models will be adequate or improved. For the maize data set, with a large number of markers, the moderate decay of singular values causes a low level of ill-conditioning. In these cases, BIR1 and BIR2 seemed to give slightly better predictions than BL, BIRR, and BAI. On the other hand, the wheat data set had a greater level of ill-conditioning, with several outliers and a drastic decay of singular values. BIRR, which assigns a constant prior variance (as standard Bayesian ridge regression), tends to over- or undershrink; this would favor markers with small singular values while penalizing markers with large singular values.

## Appendix

Recalling that model (1) is represented as a deviation from the overall mean μ, closed form distributions can be derived from their joint distributions (15), (16), (18), and (19). The conditional distribution of *μ* is univariate normal:

$$p\left(\mu \text{|}y,\beta ,\sigma_{\varepsilon }^{2}\right)\propto N(\mu \text{|}\frac{1}{n}\overset{n}{\underset{i=1}{\sum }}(y_{i}-X_{i}^{\prime }\beta ),\frac{1}{n}\sigma_{\varepsilon }^{2})$$

The conditional distribution of $\sigma_{\varepsilon }^{2}$ is:

$$p\left(\sigma_{\varepsilon }^{2}|d,b,\sigma_{b}^{2},\upsilon_{e},Sc_{e}\right)=\chi^{-2}\left(\sigma^{2}|n+\upsilon_{e},{\left(d-\mathrm{Sb}\right)}^{'}\left(d-\mathrm{Sb}\right)+Sc_{e}\right)$$

(small values are given to $\upsilon_{e}=0.0001,Sc_{e}=0.0001$).

Specifically for the BIRR model, the conditional distributions for $\sigma_{b}^{2}$ and $Sc_{b}$ are:

$$p(\sigma_{b}^{2}|d,b,\upsilon_{b},Sc_{b})=\chi^{-2}\left(\sigma_{b}^{2}|n+\upsilon_{b},b^{'}b+\upsilon_{b}Sc_{b}\right)$$

$$p\left(Sc_{b}|d,b,\sigma_{b}^{2},\upsilon_{b}\right)=Ga\left(Sc_{b}|\frac{\upsilon_{b}}{2}+1,\frac{\upsilon_{b}}{\sigma_{b}^{2}}\right)$$

For the BAI model, the conditional distribution of $\sigma_{b_{i}}^{2}$ is:

$$p(\sigma_{b_{i}}^{2}|d,b_{i},\sigma_{\varepsilon }^{2},Sc\text{)}=IG\left(\sigma_{b_{i}}^{2}|\frac{\nu +1}{2},\frac{\nu Sc+b_{i}^{2}}{2}\right)$$

The Bayesian model is completed by giving *υ* a value of 3 to avoid infinite variance in the distribution of $\sigma_{b_{i}}^{2}$. The conditional distribution of Sc is

$$p\left(Sc|d,b,\upsilon ,\sigma_{b}^{2}\right)=Ga\left(Sc|\frac{n\upsilon }{2},\left(\frac{\upsilon }{2}\right)\overset{n}{\underset{i=1}{\sum }}\frac{1}{\sigma_{b_{i}}^{2}}\right)$$

For BIR 1 and BIR2, the conditional distributions of *ϕ* $Sc_{\phi }$ are:

$$p(\phi |d,b_{i},\upsilon_{\phi },Sc_{\phi }\text{)}=IG\left(\phi |\frac{\upsilon_{\phi }+n}{2},\frac{b^{\prime }\text{Ω}b+\upsilon_{\phi }Sc_{\phi }}{2}\right)$$

$$p\left(Sc_{\phi }|d,b,\phi ,\upsilon_{\phi }\right)=Ga\left(Sc|\frac{\upsilon_{\phi }}{2}+1,\frac{\upsilon_{\phi }}{2\phi }\right)$$,

respectively.

For BIR1, ***Ω*** is a diagonal matrix of order *n* × *n*, with elements in the diagonal $\frac{1}{i^{-1-2\alpha }+h}$ for *i*=1,2,…,*n*. For BIR2, ***Ω*** is equal to matrix $S^{-1}$. For BIR1, the default value of smoothing parameter *h* is $\frac{1}{s_{r}}$.
